# Closing the loop: universal regeneration and upcycling of spent lithium-ion battery cathodes

**DOI:** 10.1093/nsr/nwaf445

**Published:** 2025-10-31

**Authors:** Xiaofeng Li, Xiaoyu Shi, Sang-Young Lee, Zhong-Shuai Wu

**Affiliations:** State Key Laboratory of Catalysis, Dalian Institute of Chemical Physics, Chinese Academy of Sciences, China; State Key Laboratory of Catalysis, Dalian Institute of Chemical Physics, Chinese Academy of Sciences, China; Department of Chemical and Biomolecular Engineering, Yonsei University, Republic of Korea; State Key Laboratory of Catalysis, Dalian Institute of Chemical Physics, Chinese Academy of Sciences, China

The direct regeneration of lithium-ion batteries (LIBs) has emerged as a state-of-the-art strategy in the recycling field, attracting growing attention from both academia and industry due to its advantages of minimal pollution, low energy consumption and high efficiency [1]. To further ensure that regenerated cathodes can satisfy the increasing performance demands of LIBs [2], researchers have proposed the concept of ‘upcycling’. This approach not only repairs degraded materials, but also tailors their intrinsic properties to deliver electrochemical performance comparable to, or even exceeding, that of fresh commercial cathodes, thereby laying a scientific foundation for sustainable energy storage.

In a recent study, Ji *et al.* regenerated spent LiMn₂O₄ (S-LMO) by using a conventional high-temperature solid-state repair and an innovative instant Joule heating method, respectively, yielding repaired LiMn₂O₄ via solid-state treatment (R-LMO-SS) and via Joule heating (R-LMO-JH) [3]. Leveraging the unique characteristics of Joule heating, they further upcycled S-LMO into high-performance cathodes such as LiNi_0.5_Mn_1.5_O_4_ (U-LNMO) and Li_1.2_Ni_0.2_Mn_0.6_O_2_ (U-LRM) (Fig. 1). Importantly, this work underscored that the core of direct upcycling lies in constructing a feasible phase-evolution pathway between the spent and target cathodes, which enables precise structural upcycling without complete cathode decomposition. This principle provides a foundation for understanding the paradigm shift introduced by direct recycling technology. The electrochemical performance of these upcycled materials surpassed those of their commercial counterparts (C-LMO, C-LNMO and C-LRM) without any additional doping or coating.

**Figure 1.**
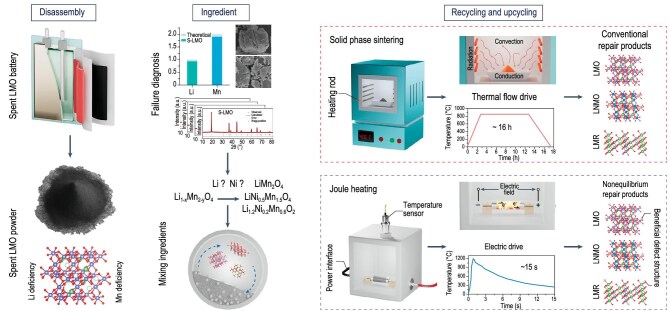
Overall experimental design of this protocol. The main experimental process includes the following: (i) disassembling spent LIBs to collect and pretreat the spent cathode materials; (ii) calculating the required additives on the basis of failure analysis results and the target product composition; and (iii) selecting an appropriate method for direct recycling or upcycling [3].

Comparative analysis highlighted the advantages of the instant Joule heating method over conventional repair, including a significantly reduced lithium supplement requirement and much shorter processing times. Structural characterizations further confirmed the effectiveness of this approach: Rietveld refinement of XRD patterns showed the full recovery of lattice parameters, while high-resolution TEM revealed the absence of lithium-deficient Mn₃O₄ phases and a uniformly restored spinel framework. In theory, Joule heating is an inherently non-equilibrium synthesis process [4], driven by its ultrafast heating rate [5]. This process induces abundant structural defects, a higher concentration of oxygen vacancies and a compositional Ni gradient within cathode particles [6]. These features contributed to the superior rate capability and cycling stability of R-LMO-JH, U-LNMO and U-LRM.

Another notable contribution of this study is the establishment of a comprehensive, end-to-end protocol for solid-phase regeneration and the upcycling of cathodes. The protocol begins with LIB discharge and includes the cell disassembly, pretreatment, characterization, repair and upcycling of the spent cathode, followed by detailed evaluation of the regenerated materials. Each stage specifies precise procedures, durations, safety considerations, reagent information and the rationales for critical steps, accompanied by diagnostic analyses and corresponding remedies.

In summary, at the material level, Ji *et al*. applied the instant Joule heating technique to upcycle S-LMO, significantly improving the electrochemical performance of the repaired materials by introducing structural defects. Building on this foundation, they provided a comprehensive blueprint for the entire solid-phase recycling process, thereby establishing a foundational paradigm for future battery recycling. At the application level, the integration of advanced technology in this work has substantially minimized resource waste, shortened repair time and enhanced overall efficiency. With further refinement, this recycling paradigm is expected to offer considerable benefits for future industrial-scale implementation.

## References

[1] Wang J, Zhang Q, Sheng J et al. Natl Sci Rev 2022; 9: nwac097. 10.1093/nsr/nwac097 35992232 PMC9385464

[2] Zhang Y, Hao S, Pei F et al. Natl Sci Rev 2024; 11: nwae254. 10.1093/nsr/nwae254 39184135 PMC11344168

[3] Ji H, Wang J, Qiu X et al. Nat Protoc 2025; doi: 10.1038/s41596-025-01234-9. 10.1038/s41596-025-01234-9 40825875

[4] Dong Q, Hu S, Hu L. Nat Chem Eng 2024; 1: 680–90. 10.1038/s44286-024-00134-1

[5] Zhu W, Zhang J, Luo J et al. Adv Mater 2023; 35: 2208974. 10.1002/adma.202208974

[6] Guo Z, Jiang H, Sun X et al. Adv Energy Mater 2024; 14: 2302484. 10.1002/aenm.202302484

